# DNA extraction protocols for animal fecal material on blood spot cards

**DOI:** 10.1371/journal.pone.0313808

**Published:** 2025-05-12

**Authors:** Ann-Katrin Llarena, Thomas H. A. Haverkamp, Wenche Støldal Gulliksen, Kristin Herstad, Arne Holst-Jensen, Eystein Skjerve, Lisbeth Rannem, Sabrina Rodriguez-Campos, Øivind Øines

**Affiliations:** 1 Department of Paraclinical Sciences, Faculty of Veterinary Medicine, Norwegian University of Life Sciences, Ås, Norway; 2 Department of Animal Health, Welfare and Food Safety, Norwegian Veterinary Institute, Ås, Norway; 3 Department of Analysis and Diagnostics, Norwegian Veterinary Institute, Ås, Norway; 4 Department of Companion Animal Clinical Sciences, Faculty of Veterinary Medicine, Norwegian University of Life Sciences, Ås, Norway; 5 Department of Production Animal Clinical Sciences, Faculty of Veterinary Medicine, Norwegian University of Life Sciences, Levanger, Norway; 6 Nord-Trøndelag Health Study, HUNT, Norwegian University of Science and Technology, Ås, Norway; Washington State University - Spokane, UNITED STATES OF AMERICA

## Abstract

**Background:**

Collecting fecal samples using dry preservatives is an attractive option in large epidemiological studies as they are easy to use, cheap and independent of cold chain logistics. Here, we test four DNA extraction methods with the aim of identifying an efficient procedure to extract high-quality DNA from fecal material of canine, sheep, equine, bovine, and pig collected on dry blood spot cards, with the goal of generating good quality shotgun metagenomics datasets. Further, the suitability of Illumina shotgun metagenomic sequencing at 20 million paired-end (PE) read depth per sample was assessed on its ability to successfully characterize the taxonomic and functional aspects of the resulting fecal microbiome.

**Methods:**

DNA was extracted from pig feces and mock communities collected on blood spot cards using four DNA extraction methods; two different methods of the QIAsymphony® PowerFecal® Pro DNA Kit, the ZymoBIOMICS™ DNA Miniprep Kit, and the MagNA Pure 96 DNA and Viral NA Small Volume Kit. Possible extraction bias was controlled by amplicon sequencing of mock communities. Fecal samples from canine, sheep, equine, bovine, and pig were thereafter subjected to the best performing DNA extraction method and shotgun metagenomic sequencing to determine sequencing efforts for functional and taxonomic analysis.

**Results:**

The four DNA extraction methods demonstrated similar community composition in the sequenced bacterial mock community. The QIAsymphony® PowerFecal® Pro DNA Kit was identified as the DNA extraction method of choice, and the resulting DNA was subjected to shotgun metagenomic sequencing with 20million PE reads. We found that higher number of reads increased the richness of observed genera between 100,000 and 5 million reads, after which higher sequencing effort did not increase the richness of the metagenomes. As for functional analysis, the number of low abundance functions in the metagenomes of the animals’ feces increased with sequencing depth above 20 million PE reads.

**Conclusion:**

Our experiments identified several methods suitable for extracting DNA from feces collected on blood spot cards. The QIAGEN’s Blood and Tissue kit on the QiaSymphony platform fulfilled the criteria of high yield, quality, and unbiased DNA, while maintaining high throughput for shotgun metagenomic sequencing. A sequencing depth of 20 million PE reads proved adequate for taxonomic estimations and identifying common functional pathways. Detecting rarer traits, however, requires more sequencing effort.

## Introduction

The microbial community in the gastrointestinal tract plays an important part in physiology and health of animals and humans. The gut microbes closely interact with the host’s epithelial cells and are important for digestion, immune response, neuro-gastro pathways, and development of metabolic and inflammatory illnesses [1–3]. The intestinal tract can also harbor numerous animal and human pathogens and antimicrobial resistance (AMR) genes and bacteria transmissible to humans, animals, and the environment [4–7], constituting a possible public health risk. Therefore, the microbial community in the animal gut has been under scrutiny in numerous studies [8–14].

Using feces as a proxy for gastrointestinal luminal content is a simple and non-invasive method to collect samples and animal owners can easily collect feces. Feces is discharged to the environment, and animal and human pathogens with a fecal-oral route of transmission makes feces relevant to study in a One Health context [7]. However, different collection methods, transport, and storage time and conditions can affect sample integrity [15–19], and thus the inference of the microbial community composition and nucleic acids profile, albeit the variation observed among individuals is expected to exceed the bias induced by various methods and approaches for sampling [17,19,20]. Sampling, transport, and storage procedures should nevertheless be selected to minimize post-collection bias in microbial community composition. The presumptive gold standard is considered to be rapid freezing [20,21], or the use of liquid preservatives such as 95 percent ethanol, DNA/RNA Shield (Zymo Research), and RNAlater (ThermoFisher). These methods are impractical in large-scale field research where the collection method must be simple, independent of cold chain logistics, and often have budget constraints [22]. Dry collection methods involve using containers, material or devices that preserve the fecal samples in a dry state. These can be dried blood spot (DBS) cards made of protein-binding cellulose, which have been in use in diagnostics since 20^th^ century [23], and more recent materials made of papers treated with chemicals to lyse microorganisms and bind proteins and nucleic acids, such as occult blood test cards (FOBT) and Flinders Technology Associates (FTA) cards (Whatman PLC, UK). These cards are designed to keep the sample dry and stable during transportation and storage and are appealing choices for sampling fecal material in large field investigations since they are simple to use, easily transportable, can be kept at room temperature, and do not require any additional preservative chemicals. Dry preservatives preserve human feces efficiently [18,19,24], including during long term storage [25,26], but they allow less sample material to be collected and makes it impossible to measure its initial weight (as reviewed by [27]). A few studies have used dry cards to collect fecal material from primates [28], birds [29], and cats [30] intended for microbiota profiling using amplicon sequencing, finding that dry preservatives preserved DNA, albeit with low yield. This limitation must be considered when choosing library protocols, sequencing technique, and choice of bioinformatics [30].

The Trøndelag Health study (HUNT) One Health is a large cohort study that collected feces and health data from ~3000 animals (canine, sheep, equine, bovine, and pig) [31]. The animal owners collected fecal samples on DBS cards, air-dried these, and sent them to a laboratory for storage. The goal was to generate shotgun metagenomic datasets suitable for functional and taxonomic characterization to the greater research community. Adequate yield and high-quality DNA are required, as low DNA levels can result in higher biases in GC content, k-mers, or target (e.g., 16S rRNA) gene compositions [32]. A critical lower amount of environmental DNA suitable for shotgun metagenomics has been reported to be 10pg of DNA (32). The most suitable extraction method for DNA from animal feces on DBS cards is not known. Taxonomic and functional characterization of fecal metagenomes using amplicon sequencing are available for all relevant species, but few studies use shotgun metagenomics to characterize the fecal metagenome from animals (as reviewed in [33]).

The main aim of the present study was to identify a suitable and common procedure for high-throughput acquisition of shotgun metagenome data from dried animal feces collected on DBS cards. For this, the chosen extraction method should achieve sufficient yield and high-quality DNA, minimized bias in metagenome DNA profiles, and provide sufficient data to allow for the desired level of characterization of taxonomic and functional properties present in the fecal microbiome(s). The chosen procedure was to be successively applied to all animal feces samples collected in the HUNT One Health study.

## Materials and methods

### Materials

#### Animal feces on DBS cards.

**Fig 1:**
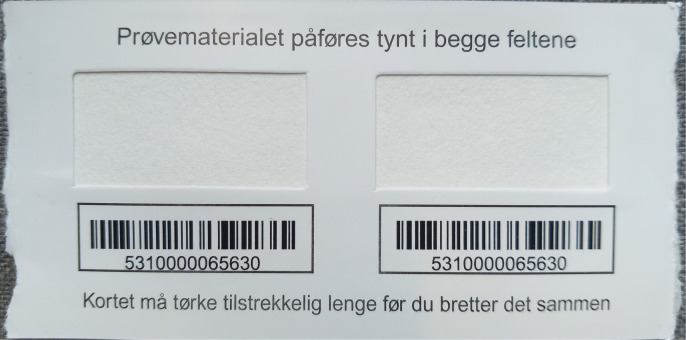
Picture of an empty DBS card with two fields of 170mm*350mm each, equaling an area of ~ 12 cm2 available for sampling.

The samples in HUNT One Health were collected by untrained animal owners, based on instructions on paper and video. The owners smeared animal fecal material on two sampling fields of 1.7 x 3.5 cm DBS filter paper pieces contained in a customized wrap-around card (Lipidx, Oslo, Norway), hereafter referred to as “DBS card” (Fig 1). We therefore used the same type of DBS filter paper to allow simulation of samples in the HUNT One Health project. Fecal samples of healthy canines, sheep, equines, bovines, and pigs were collected and smeared as a thin layer on the DBS cards, dried for at least two hours and stored at -20°C before pieces of this material were subjected to DNA extraction. Upon extraction, the DBS cards were taken out from the freezer and thawed. Four 8-mm diameter circles were aseptically clipped from each DBS card with a single-use biopsy puncher (cat number 273693, Kruuse Norway, Drøbak, Norway). The operators visually ensured similar and representative sections of the DBS with evenly spread fecal material to be selected for sampling. The paper circles were transferred to PowerBead Pro Tubes (2 ml) (cat. no. 19301, QIAGEN) containing pre-loaded homogenization beads.

#### Mock and blank samples.

To prepare positive and negative template controls, four 8-mm diameter paper discs were aseptically excised from a clean DBS and deposited in a PowerBead Pro tube. Positive controls were spiked with 75 µl of the standard microbial community II from Zymobiomics (Cat. No. D6310, Zymo Research Corporation, USA), hereafter referred to as “mock” samples. This mock community consists of known quantities of Gram-negative and Gram-positive bacteria together with yeast with varying sizes and cell wall composition. The theoretical composition in terms of 16S rRNA gene abundance as given by the producer, calculated from theoretical genomic DNA composition with the following formula: 16S rRNA gene copy number = total genomic DNA (g) × unit conversion constant (bp/g)/ genome size (bp) × 16S copy number per genome, is 95.9% *Listeria monocytogenes*, 2.8% *Pseudomonas aeruginosa*, 1.2% *Bacillus subtilis*, 0.069% *Escherichia coli*, 0.07% *Salmonella enterica*, 0.012% *Lactobacillus fermentum,* 0.00089% *Enterococcus faecalis*, and 0.000089% *Staphylococcus aureus* (Zymobiotics Research Corporation, USA). Negative controls were DBS paper only, subjected to the same buffers and procedures as the samples containing fecal material, and is hereafter referred to as “blanks”.

#### Experimental set up.

**Fig 2:**
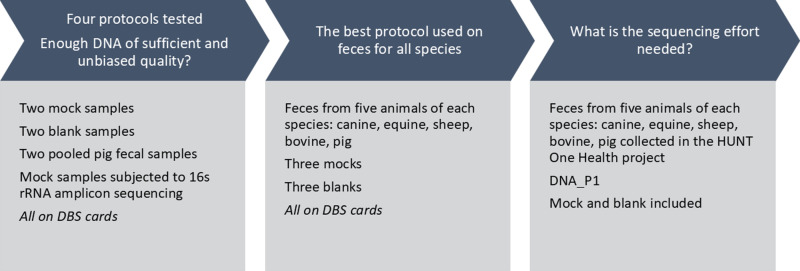
Experimental set up to identify a suitable DNA extraction and purification protocol for DBS. The extraction methods DNA_P1, DNA_P2, DNA_P3 and DNA_P4 protocols were used to extract DNA from mocks, blanks and pig feces and the resulting mock DNA was subjected to 16s rRNA gene amplicon sequencing using Nanopore MinION (Oxford Nanopore Technologies, Oxford UK) to assess yield and potential bias introduced in the DNA extraction. To inform on required sequencing effort, the best performing method according to amplicon sequencing results (DNA_P1), was used to extract DNA from bovine, equine, canine, sheep, and pig feces. This DNA was subjected to shotgun metagenomic sequencing using the Illumina NovaSeq platform to generate >20million PE reads from each sample.

To identify the most suitable DNA extraction method, we first evaluated four different procedures on their efficiency to purify DNA from pigs and mock community. Mock samples were prepared as described above, while pig feces were collected from two healthy adult pigs, pooled, and properly mixed before being smeared as a thin coating on DBS cards. To assess potential biases in microbial community profiles introduced by DNA extraction method, resulting DNA from mock samples were subjected to 16s rRNA amplicon sequencing using Nanopore technology, as this was a cost-effective, rapid in-house NGS method. Based on these initial findings, we selected the most suitable extraction method to assess the methods’ ability to purify whole DNA from fecal samples collected on DBS cards from the five animal species included in the HUNT One Health project—canine, sheep, equine, bovine, and pig. The DNA were thereafter subjected to shotgun metagenomic sequencing using Illumina technology, which allowed us to make decision on the sequencing efforts that provides taxonomic and functional characterization (Fig 2).

### DNA extraction

The pig feces, mock samples and blanks were subjected to DNA extraction with four different protocols: DNA protocol 1 (DNA_P1): QIAsymphony® PowerFecal® Pro DNA Kit (cat. no. 938036, QIAGEN) including six rounds of homogenization using the FastPrep-24™ Classic (MP Biomedicals, Irvine CA, USA) at 6 m/s for 60 seconds, separated with 5 minutes of resting, followed by digestion with proteinase K 600 mAU/ml (QIAGEN) prior to automated purification on the QIAsymphony® SP robot (QIAGEN). DNA_P2 was performed identical as DNA_P1, but without proteinase K treatment. DNA_P3 was ZymoBIOMICS™ DNA Miniprep Kit (Zymo Research Corp., Irvine, CA, USA) after homogenization using a FastPrep-24™ Classic at 6m/s for 60s for six rounds interspaced with 5 min resting. DNA_P4 was MagNA Pure 96 DNA and Viral NA Small Volume Kit on the MagNA Pure 96 Instrument (Roche, Basel, Switzerland) with proteinase K pre-treatment step and standard buffers using the DNA Blood ds SV Protocol optimized for double stranded DNA and Next Generation Sequencing (Table 1). Details on the DNA extraction methods can be found in “S1 Methods”. The obtained elute purity and yield was assessed, as well as its capability for high throughput, and the resulting DNA from the mock communities was subjected to amplicon sequencing targeting the bacterial 16S rRNA gene using the Oxford Nanopore MinION sequence device (Oxford Nanopore Technologies, Oxford UK) (see chapter “ Library preparation and sequencing strategies”).

**Table 1:** Overview of extraction methods tested.

Method name	Instrument platform or manual	Kit/Reagents	Elution volume
DNA_P1	QIAsymphony® SP robot	QIAsymphony® PowerFecal® Pro DNA Kit (cat. no 938036, QIAGEN) with proteinase K treatment	110 µl
DNA_P2	QIAsymphony® SP robot	QIAsymphony® PowerFecal® Pro DNA Kit (cat. no 938036, QIAGEN)	110 µl
DNA_P3	Manual	ZymoBiomics™ DNA Miniprep Kit (Zymo Research Corp., Irvine, CA, USA)	100 µl
DNA_P4	MagNA Pure 96 Instrument	MagNA Pure 96 DNA and Viral NA Small Volume Kit with proteinase K	50 µl

The best performing protocol considering DNA yield, purity, applicability for high-throughput processing, and ability for generating unbiased sequence data as assessed by 16s rRNA amplicon data, was used to extract DNA from fecal samples collected from five individuals of each species: canine, sheep, equine, bovine, and pig (in total n = 25, Fig 2). Triplicates of mock and blank samples were included in each protocol (Fig 2). The DNA purity and yield was measured and its combined potential for high throughput processing was assessed.

The DNA concentration was measured fluorometrically by Qubit™ (Qubit, Thermo-Fisher Scientific, Waltham MA, USA) using the Qubit dsDNA BR (Broad Range) Assay Kit or, if the concentration was too low to yield a result, the Qubit™ dsDNA HS (High Sensitivity) Assay Kit. DNA integrity was assessed by agarose gel electrophoresis either in house or by the sequencing provider, while DNA purity was assessed using a Nanodrop Spectrophotometer (Thermo-Fisher Scientific, Waltham MA, USA) at absorbance wavelength ratio 250nm/230nm against 260nm. A ratio > 1.8 was considered pure. Descriptive statistics for each extract over the four extraction methods were calculated in STATA v.15.1 SE for Windows (StataCORP LLC, Texax, USA) and visualized with normalized log10 values using Microsoft Office 2016 Win 32 Excel (Microsoft, California, USA)

To determine sufficient sequencing efforts needed for functional and taxonomical characterization of the fecal metagenomes in the HUNT One Health project, canines, sheep, equines, bovines, and pigs’ fecal samples collected on DBS cards between 2017 and 2019 were extracted using DNA_P1 and subjected to shotgun metagenomic sequencing using Illumina technology (Fig 2). Together with one mock (described above) and a blank sample, the DNA was submitted to sequencing (~20 million PE reads) via library preparation using the ThruPLEX library preparation kit (Takara, Shiga, Japan), and 150 bp PE sequencing on the Illumina NovaSeq platform (see chapter “Sequencing strategies”).

### Library preparation and sequencing strategies

#### Amplicon sequencing.

DNA extracted from mock samples using DNA_P1 through DNA_P4 was subjected to 16S amplicons sequencing using the nanopore sequencing device MinIon (Oxford Nanopore Technologies, Oxford, UK). The amplicons were generated from the DNA-templates with a PCR using the universal primers 27F/S-D-Bact-0008-c-S-20 and 1492R/S-D-Bact-1492-a-A-22 for long read characterization of bacterial communities with a long-read NGS protocol [34] using a recombinant DNA polymerase (EP0402, ThermoFisher Scientific, Waltham, USA). Amplicons were purified using Macherel-Nagel Nucleospin Gel and PCR cleanup-kit (Düren, Germany), quantified with Qubit 1x HS DNA assay (ThermoFisher Scientific), before up to 440 ng of purified amplicons were subjected to nanopore library preparations using the rapid barcoding kit (SQK RBK004, v RBK_9054_v2_revM_14Aug2019) according to manufacturer’s description with one modification: double volumes were applied to allow top up of the flow-cell (FLO-106D) after a period of sequencing. In total, the Minion flow-cell ran for 28 hrs.

To obtain FASTQ files, the fast5 data was base called using Guppy (version 6.5.7) with default settings (Oxford Nanopore). The output file from Guppy was used for quality control with Nanoplot (version 1.33.1 [35]). A total of 2.4 x 10^6^ reads were produced with a median quality Phred score of 12. The fastq reads were split on adapter using the script duplex_tools (ONT) to generate simplex reads. All FASTQ files were concatenated and subsequently demultiplexed with qcat (version 1.1.0; https://github.com/nanoporetech/qcat) to generate a single FASTQ file per sample with the following settings: minimum read length 50 bp, trim adapters and barcode sequences, and detect-middle. Next, we used NanoFilt (version 2.8.0) to select only reads with an average Phred quality score of 9 or higher with a read length between 1450 and 1650 bp, matching the size of the 16s rRNA [35]. Classification of the estimated number of reads based on relative abundance was performed with the tool EMU, using a minimum abundance of 0.0001 with the EMU 16s rRNA classification database as well as the SILVA database [36]. The EMU results were imported into R-studio (version 2022.07.01) to visualize the diversity of the samples. The following packages were used in R-version 4.2.1: tidyverse (v1.3.2); ggplot2 (v3.5.1); microViz (v0.12.4)[37].

#### Shotgun metagenomic sequencing.

Library preparation and whole genome Illumina shotgun metagenomic sequencing was performed by BGI Tech Solutions (Hong Kong, China). Libraries were prepared using the ThruPLEX® DNA-seq by Takara (cat. no. R400674, Takara BioInc, Europe), which can generate libraries from as little as 50 pg of DNA. The concentration and fragment length of the resulting libraries were quality checked on a 2100 Bioanalyzer (Agilent, Santa Clara, US) with an in-house qPCR to meet the criteria of Illumina Novaseq sequencing requirements. The libraries were thereafter sequenced on the Novaseq 6000 using the 150 bp PE sequencing strategy, delivering at least 5Gb data per sample.

Quality control and classification of Illumina shotgun metagenomic datasets was first performed by the external sequencing provider (BGI), which delivered clean reads, from which adaptor sequences, contamination and low-quality reads had been removed using SOAPnuke software (version 2.1.0)[38]. In brief, SOAPnuke removed the entire read if it contained >25% adapter sequence, > 50% bases with a quality score < 20, or > 3% N bases. Next, duplicate reads with identical bases were removed from the data. This clean sequence data was processed using the Nextflow pipeline TALOS (https://github.com/NorwegianVeterinaryInstitute/Talos). In brief, the pipeline filters low-quality and low complexity reads and reads matching the human/phix (NC_001422) genomes [39]. The cleaned data was then subsampled with the seqtk; version 1.3 into datasets with 10k, 50k, 100k, 500k, 1Million, 5M, and 10M PE reads. Each dataset was then classified taxonomically using Kraken 2 [40], and the PlusPFP database (date: 12/9/2022; https://benlangmead.github.io/aws-indexes/k2). In addition, functional classification with SUPER-FOCUS [41] using the Diamond version 1 database with cluster size 100 (https://github.com/metageni/SUPER-FOCUS#downloading-prebuilt-databases) was performed [42]. To identify compositional differences between host species, Sourmash [43] was used to generate kmer sequence signatures from each sample. The signatures were used to generate a Jaccard distance matrix and were visualized using Sourmash plot. The taxonomic and functional classification results were imported into R studio (version 2022.07.1) running R (version 4.2.1). The r-package tidyverse (1.3.2) was used for reformatting the data and visualization of the results. Vegan (2.6–4) [44] was used for calculation of alpha-diversity metrics for both the Taxonomic and functional classifications.

## Results

To identify a suitable DNA-extraction method for the fecal material collected in the HUNT One Health project, the combined results from DNA concentration measurements, efficiency, microbial community biases introduced, and level of automation and cost of different extraction methods, were compared.

### DNA concentration and yield

**Fig 3:**
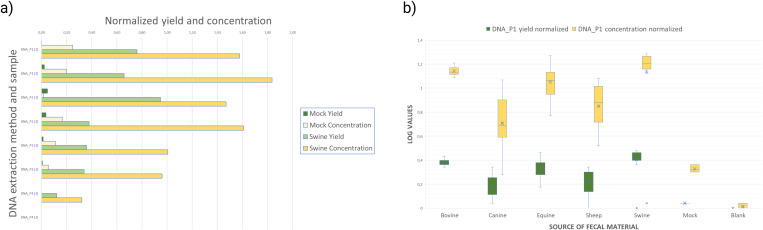
Barchart depicting the distribution of DNA yields and concentrations achieved using four different extraction protocols for pig, mocks and blanks (a) and pigs, equine, sheep, canines and bovines (b). To account for variability in the dataset and normalize the data distribution, a logarithmic transformation was applied to the values. Specifically, the transformed values were computed using the formula; *log10(Value−min + 1),* where *Value* represents the data point of interest and *min* is the minimum value within the dataset. This transformation ensured that all values remain positive and prevents computational errors associated with logarithms of zero or negative numbers. These plots provide a comprehensive view of the variability in DNA extraction efficiency among the different protocols employed.

The median DNA concentration from mock and pooled pig fecals was 0,34 (range 0,1–0,88) ng/µl and 26,8 (range 7,9–75,6) ng/µl, respectively. Blanks were always below detection limit of the Qubit assay. DNA protocols using QiaSymphony (DNA_P1 and DNA_P2) achieved the highest median DNA concentrations from pig feces (n = 2, 60,2 (range 44,8–75,6) ng/µl and 42,1 (range 36,5–47,6) ng/µl) of all methods tested (S1 Table), while DNA_P3 and DNA_P4 resulted in lower and comparable DNA concentrations (Fig 3a).

### Depiction of the mock communities

The DNA of the mock samples was extracted using protocol DNA_P1 through DNA_P4 and subjected to 16S rRNA amplicon sequencing using MinION (Oxford Nanopore Technologies). The range in the number of classified reads from each mock community was 5 885 (DNA_P4) – 216 557 (DNA_P2), of which 0–453 reads were not one of the eight bacterial species in the mock community. The average relative abundance of *L. monocytogenes* was 98.8% (SD 0,25%), which was higher than expected for the mock community (theoretical relative abundance of 95.7%) (S1 Fig). The average relative abundance of the remainder of the bacteria expected in the mock samples were 0.85% (SD 0.18%) *P. aeruginosa*, 0.32% (SD 0.07%) *B. cereus*, 0.02% (SD 0.01%) *S. enterica* and 0.01% (SD 0.01%) *E. coli.* *S. aureus, E. faecalis and L. fermentum* were not detected in any of the mock extracts. In mock extracts created using DNA_P2 (n = 1) and DNA_P3 (n = 1) and DNA_P4 (n = 2), none of the sequenced reads matched to *E. coli* nor *Salmonella*.

### Defining a suitable sequencing effort – shotgun metagenomic dataset

DNA from 35 fecal samples from the five different animal species, mocks and blanks was extracted using DNA_P1 (Fig 3b, average concentration and total yield are given in S1 Table) and subjected to deep shotgun metagenomic sequencing using the Illumina Novaseq platform. The average number of reads per fecal sample (irrespective of animal species) was 2,1 x 10^7^ PE reads [SD ± 4,5 x10^6^, range 6,0 x 10^6^–2,4 x 10^7^], and the average number of reads for each dataset over species were comparable between bovine, sheep, pig, and equine samples, while the average number of reads for canine samples was slightly lower than for the other species (Table 2, S2 Table).

**Table 2:** Sequence effort over samples.

DNA extraction method	Source	Average # PE reads [SD]	Range # PE reads
DNA_P1	All animals	2.1*10^7^ [SD ± 4471759]	6.2*10^6^, 2.4*10^7^
	Bovine	2.1*10^7^ [SD ± 3708954]	1.6*10^7^, 2.4*10^7^
	Canine	1.7*10^7^ [SD ± 6541075]	6.2*10^7^, 2.4*10^7^
	Equine	2.3*10^7^ [SD ± 2048548]	1.9*10^7^, 2.4*10^7^
	Sheep	2.1*10^7^ [SD ± 3494134]	1.6*10^7^, 2.4*10^7^
	Pig	2.1*10^7^ [SD ± 5084972]	1.2*10^7^, 2.4*10^7^

**Fig 4:**
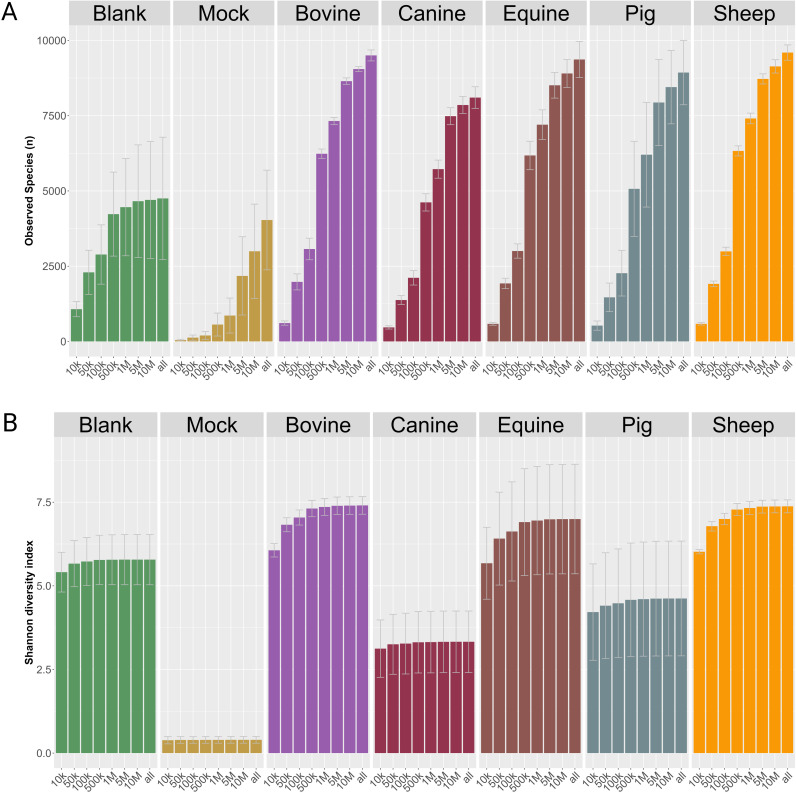
Observed species (a) and Shannon diversity (b) for all samples in relation to the subsampling depth. Each bar represents the average value for the five samples. Error bars indicate the standard deviation. As little as 5*10^4^ reads was sufficient to capture the Shannon diversity of mock samples, while blank samples Shannon diversity tapered off after 5 x 10^5^ reads. For fecal samples, the Shannon diversity was unchanged after approximately 5 million reads.

To decide on the appropriate sequencing depth needed to reliably define the functional and taxonomic composition of fecal matter of the five sampled species, the datasets were randomly subsampled at 1 x 10^4^, 5 x 10^4^, 1 x 10^5^, 5 x 10^5^, 1 x 10^6^, 5 x 10^6^ and 1 x 10^7^ reads, creating in total 560 datasets. Each dataset was thereafter taxonomically classified using the TALOS pipeline’s inbuilt Kraken2 with the standard database plus protozoa, fungi & plant (plusPFP) (Standard is: Prokaryotic RefSeq genomes, virus genomes and plasmids). The result of these analyses is presented in Fig 4. The number of observed genera (richness) increased for all datasets when more reads were analyzed, especially between 1 and 5 million reads, albeit the increase in richness tapered rapidly off for blank samples (Fig 4a). After 5 million reads the number of new observed genera per added read decreased (Fig 4a), and the Shannon diversity for all samples was unchanged for datasets larger than 5 million reads (Fig 4b). We did observe larger standard deviations in Shannon diversity for samples from pig and equine samples due to more observed genera for these samples (Fig 4b).

**Fig 5:**
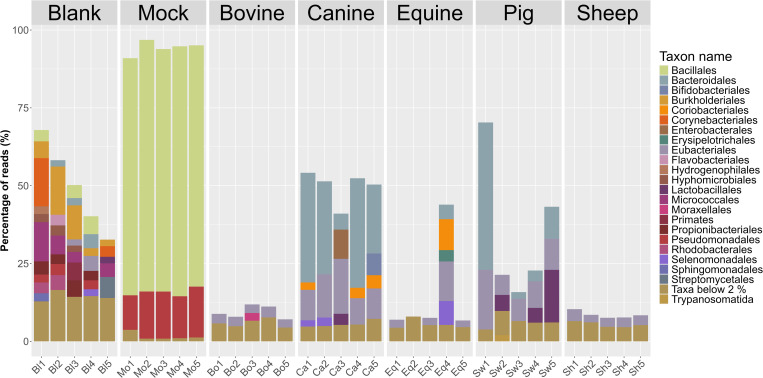
Relative abundance of orders across samples and species for the full dataset before subsampling. A low percentage of reads were classified for sheep, bovine and except for one sample, equine, while for canine and pig feces higher reads classification levels are observed. In total 66 taxa at the order level were identified with a minimum abundance of 0.1%. We only show taxa with a minimum abundance of 2% in at least one sample, all other taxa were group as “Taxa below 2%” in the legend.

Based on the full dataset, the orders Bacteroidales and Eubacteriales (previously Clostridiales) dominate most fecal samples (Fig 5). However, there are species specific differences. For instance, Lactobacillales were more abundant in several of the pig samples. Another difference between the host-species microbiomes is the fraction of classified reads in the microbiomes. For canine fecal microbiomes the classified fraction of the microbiome is much larger than for the bovine microbiomes.

**Fig 6:**
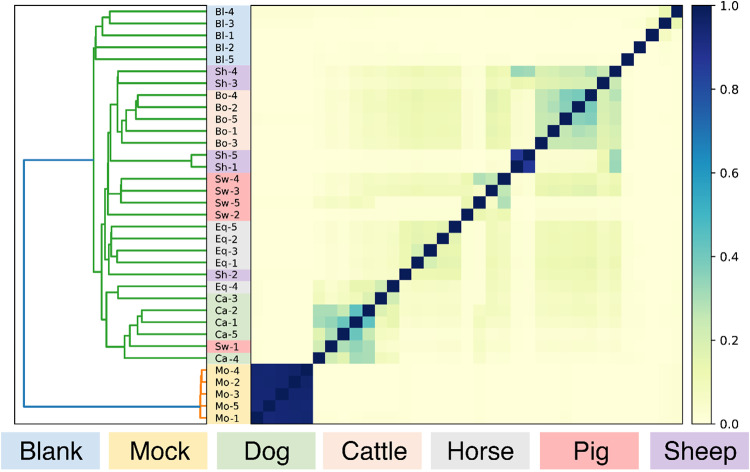
Sourmash cluster analysis of metagenomic datasets of animal’s fecal material, blanks, and mock communities. Sourmash creates a kmer signature for each of the datasets, which is used with angular similarity as a distance matrix to cluster the samples. Note that the fecal microbiomes of sheep and bovine cluster, and that mock and blank samples are outgroups of the fecal microbiomes.

Aside from taxonomic classification, DNA sequence composition based on k-mers can be used to cluster microbiome samples with the software sourmash. In this analysis, mock communities clustered together with short branch length due to their great compositional similarity (Fig 6). Animals of the same species and blanks clustered together, with longer branch lengths indicating greater variability between individual animals and blank samples due to random effects (Fig 6).

The functional potential present in the microbial populations was characterized through analyses of the Illumina shotgun metagenome data. The number of reads functionally classified per sample varied depending on the sample type (S2 Table). Blank samples had on average 1,5 million classified reads (SD ± 2,5 million), while mock samples had 12,5 million classified reads (SD ± 1,2 million). The number of classified reads for the fecal samples from the different animal species were 4,2 million (SD ± 1,1 million) for sheep, 4,8 million (SD ± 0,8 million) for bovines, 4,9 million (SD ± 1,2 million) for equines, 5,7 million (SD ± 1,7 million) for pigs, and 6,3 million (SD ± 2,1 million) for canines.

**Fig 7:**
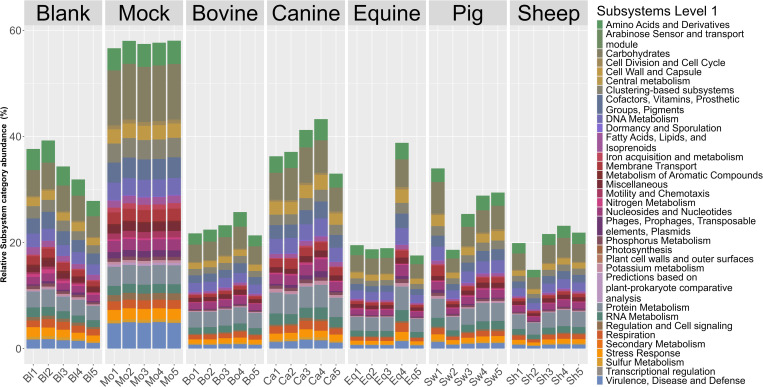
Overview of functional subsystem groups for all samples. Relative abundance of reads classified to different SEED subsystem level 1 categories. Note that bovine, sheep and equine have the lowest levels of classified reads.

The mock and canine samples had the highest percentages of reads matching sequences in the SEED database (57,6% (SD ± 0,6%) and 38,2% (SD ± 4,1%), respectively), while the sheep samples had the least reads matching (20,3% (SD ± 3,3%)) (Fig 7). The SEED subsystems with most reads assigned regardless of sample type classified to the metabolism of carbohydrates (5,0% (SD ± 2,5%)), followed by protein metabolism (3,2% (SD ± 0,5%)) and amino acids and derivatives (2,8% (SD ± 0,9%)) (Fig 7). On average, the three most abundant SEED functions for all samples combined were TonB-dependent receptors (1,3% (SD ± 1,4%)), β-galactosidase (0,8% (SD ± 0,7%)) and multi-antimicrobial extrusion proteins (MATE) (0,7% (SD ± 0,3%)). These are functions resembling uptake (TonB-dependent receptors) and export (MATE) functions, while Beta-galactosidase plays an essential role in carbohydrate metabolism. The blank samples had the lowest average number of observed metabolic functions (10.7% (SD ± 4.5%)), while bovine samples had the highest number of metabolic functions (20.9% (SD ± 1.9%)).

**Fig 8:**
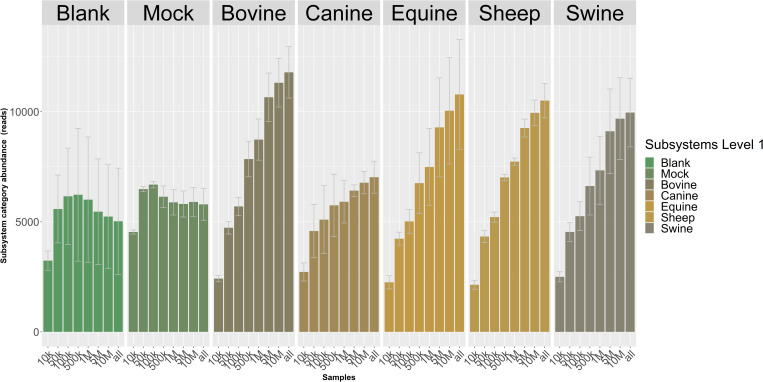
Overview of the number of functions that have less than 10 reads assigned for different sizes of the metagenomic communities. Each bar represents the average value for the five samples. Error bars indicate the standard deviation.

Using the subsampled datasets at 1 x 10^4^, 5 x 10^4^, 1 x 10^5^, 5 x 10^5^, 1 x 10^6^, 5 x 10^6^ and 1 x 10^7^ reads, the 560 datasets were subjected to functional classifications. The number of metabolic functions increased with increased sequencing depth and did not taper off for any of the fecal samples nor the mock samples. For the blanks we observed a levelling off in the number of metabolic functions after 5 million reads, due to the limited number of reads produced in these samples (Fig 8). To explore the dynamics of observed functions with sequencing depth in detail, we divided the metabolic functions in two groups: 1) infrequent functions, i.e., functions with less than 10 reads, and 2) frequent functions that have 10 or more reads. We then determined for the different subsampling depths the number of infrequent functions (Fig 8). That shows that the number of infrequent functions increases with sequencing depth for the animal fecal microbiomes, while this was not the case for the blank and mock samples. This indicates that the sequencing depth was sufficient to observe most functions for the blanks and mocks. In contrast, the frequent function class increased with increasing sequencing depth for all sample types (data not shown), since functions move from the infrequent group to the frequent group with increasing sequencing depth. These results indicate that our sequencing depth for metabolic functions was not saturated for the animal fecal microbiomes.

One group of metabolic functions that is of broad interest are antimicrobial resistance genes. In the SEED classification system, we find these genes under the subsystem level 2 category: “Resistance to antibiotics and toxic compounds”. Within that category, we find that several classes dominate in the animal fecal microbiomes (S2 Fig). Those are cobalt-zinc-cadmium resistance, copper homeostasis, multidrug resistance efflux pumps, and resistance to fluoroquinolones. Among the minor classes present in fecal microbiomes are beta-lactamases, enzymes that can breakdown a variety of antibiotics. This class of enzymes had the highest abundances in both the blank (482 reads per million reads (RPM) (SD ± 246 RPM)) and mock (431 RPM (SD ± 14 RPM)) samples. Beta-lactamases were found in the highest levels from canine samples (355 RPM (SD ± 35 RPM)) and in low levels from sheep feces (150 RPM (SD ± 37 RPM) (S2 Fig).

As we are interested in understanding the ability to detect AMR genes in our metagenomes, we selected one of the more abundant beta-lactamase gene functions and analyzed the abundance with increasing sequencing effort. The function we picked was: “Beta-lactamase_class_C_and_other_penicillin_binding_proteins”. This function could only be detected in the animal fecal microbiomes with 100 reads or more when the sequencing effort was above 1 million reads (S3 Fig). For other functions, the sequencing effort had to be higher. For example, the function “Regulatory_protein_BlaR1” was only detected in two samples, with more than 100 reads, when using all the reads sequenced. This shows that our sequencing effort was insufficient to detect low abundant AMR genes.

## Discussion

In this study, we identified an efficient, high-throughput DNA extraction method that gives high yield, quality, and unbiased DNA from canine, sheep, equine, bovine, and pig feces spread on DBS cards. Furthermore, upon deep shotgun metagenomic sequencing, the resultant DNA was successfully used as a template for metagenomic analysis to determine the taxonomic and functional composition of the metagenome. To boost sensitivity of rare functional traits, increased sequencing effort is needed. Nevertheless, the data is usable for various scientific studies, including analyses of microbiota and AMR in Norwegian production and companion animals, that potentially provide a path towards sustainable re-use of metagenome sequence datasets for One Health scientific discoveries.

Ruminants and horses are fore- and hindgut fermenters, dependent on a well-functioning gut microbiota in the rumen and ceca for the digestion of insoluble grass feeds. The microbiota in turn provides their hosts with nutrients such as short chain fatty acids and proteins [45–47]. For monogastric animals, the intestinal microbiota contributes to gastrointestinal health. Severe disturbances to the composition of the gut microbiota (dysbiosis) is associated with progression and development of gut disease such as acute hemorrhagic diarrhea syndrome [8] and colonic cancer [48,49]. Studies on the composition of gut microbiomes of these five animal species are therefore important.

HUNT One Health is the first study that used DBS cards to collect fecal material from canines, sheep, equines, bovines, and pigs intended for metagenomic analysis. As DBS cards lack DNA-stabilizing chemicals, concerns were raised on the suitability of these cards to preserve the fecal samples, and the microbial DNA contained within feces, well enough to allow for reliable qualitative and semi-quantitative metagenomic characterization. Especially degradation of genetic material during storage and transport was a concern. In addition, the cards were not designed to limit cross-contamination between samples and can only collect a small volumes or mass, possibly reducing the usability of fecal samples for metagenomics. When these challenges are acknowledged during planning, they can be addressed. For instance, the project coordinators provided the owners with specialized pouches with barcodes to protect each card and eliminate cross-contamination. These pouches also contained a small desiccant (silica gel) to help dry the samples. A Takara library preparation specialized for samples of low biomass was used to overcome the challenge of low sample volume. With time, as technology advances, the need for higher DNA amounts for sequencing reaction are also expected to decrease.

DNA extraction can induce various types of bias affecting the inference of the metagenome composition, as shown in several studies. The main goal for this study was therefore to identify a DNA extraction method suitable to extract DNA from 3000 fecal samples collected on DBS cards. The final method had to be suitable for metagenome DNA extraction from feces samples of all five animal species. The efficacy of a DNA extraction method is determined by the amount and type of sample that can be processed properly and the capacities of the extraction kit to cope with chemical and physical inhibitors, including the filter paper itself, the requirements for equipment and training of personnel, etc. (reviewed in [50]). The DBS sample approach is not standardized for amounts of feces on the cards, so irregular amounts of feces will result in variance in the quantity of input material in the DNA extraction. We tried to limit this by using four discs and trained personnel. An alternative that would increase the throughput time, would be to measure the fecal material prior to processing via gravimetric measurements. A further problem occurs when extracting DNA from fecals from different animal species; incompletely digested dietary material such as fibers in stool differs between individuals and groups such as between herbivores and omnivores [51,52], resulting in inconsistent amounts of microbes available from the same amounts of feces from different animals. Also, the protein and fatty content of the fecal, as well as more global chemical properties such as pH, vary and can affect the extraction protocol’s overall efficiency. A successful depiction of the microbial community of feces will also be dependent on the community itself, and that varies between species and individuals [53]. Ruminants, hind-gut fermenters and monogastric animals exhibit significant differences in their digestive systems. Ruminants possess a multi-chambered stomach, including the reticulum, rumen, omasum, and abomasum, which enables them to digest plant materials through fermentation. Monogastric animals have a simple stomach structure with a single compartment. These different structures will harbor microorganisms that perform digestive tasks on different materials. Therefore, the microbiome differs significantly between species [53,54]. Some of the microbes may be protected from lysing enzymes by encapsulation in various fecal materials. In such cases rigorous crushing could aid in releasing the microbes into solution and lysing. Some spore-forming parasites like *Eimeria*, have though walls preventing DNA from being released into solution unless cells are first rigorously treated. DNA extraction targeting “all” organisms present in stools, would very likely benefit from an efficient homogenizing bead-beating steps [55]. Finally, for the HUNT One Health study, the sampling was performed by the animal owners, who had received no prior training and had to follow instructions provided on paper and online video. It is reasonable to assume that they performed the sampling, smearing of samples on faecal cards and successive air-drying variably. Large variations were observable between samples derived from the same animal species, and some cards had obviously not been sufficiently dried before inserting them in the closed pouches for storage and shipping to the HUNT centre. All these variables make quantitative metagenomics, especially for rarer traits, challenging, and this dataset is therefore most suited for qualitative metagenomics.

The mock community in the microbial community standard can be used to assess the quality of extraction, possible bias, and errors in the process using 16s rRNA amplicon sequencing with long-read Nanopore technology. Of the eight microbes from the mock community, all bacteria present at 0.069% or more were identified in one or more of the samples. The relative abundances of the extracted DNA deviated somewhat from the theoretical composition of the mock community, most notably with a slightly higher abundance of *L. monocytogenes* and *B. subtilis* and lower abundance of *P. aerginosa* and *E. coli*, i.e., a small shift towards Gram-positive bacteria. This could indicate a bias in either the extraction method, amplification process, sequencing or bioinformatic pipeline. All four protocols used in this study included a homogenizing step of six minutes bead-beating on the efficient FastPrep-24 instrument. Zhang and colleagues (2021) found that increasing bead-beating time, especially over four towards nine minutes, correlated with a higher abundance of Gram-positives and reduced recovered Gram-negative bacteria [56]. Further, nanopore sequencing is known to be error-prone, and as the existing OTU-based approach require at least 97% sequence identity, such sequencing have been considered unsuitable for taxonomic classification of MinION™ reads [34].

With the start of the metagenomics era, the need for the inclusion of control samples arose, followed by discussions on how to deal with sequence data from such controls and the choice of appropriate filter methods to remove sequences that stem from potential contaminants. Negative (blank) controls have been used in many studies, to subsequently identify and remove taxa present in those controls as potential contaminants, based on the assumption that prevalence of contaminants will naturally be higher in controls than in the samples because of absence of competing DNA [57]. However, for this approach to work the input biomass of all sequenced samples must be equal [58]. This is a problem for blank samples, which usually contain very minute amounts of DNA. In our hands, sequencing the blanks generated enough reads which were used for community analysis (S2 Table), and the diversity analysis and taxonomic classification indicated a blank community consisting of many different taxa. However, most of these taxa were present in small number of reads only (<50 reads) and the classifications are therefore possibly spurious. However, some of the taxa are most certainly present (e.g., Burkholderiales, Micrococcales), which makes sense as our blanks were neither sterile nor aseptically handled and were included to depict what was in the paper used to collect the fecal samples. Further, the bioinformatic analysis did not apply a lower threshold for reporting taxa, so all classified reads will be reported, regardless of how rare they were, explaining the observed richness of these samples. The community of the blanks differ significantly from the fecal samples and the mock communities, demonstrating that neither the collection method nor the kitome present influenced the results from the fecal community.

In general, our experiments found several methods to be useful, and we were able to perform an informative decision on which method will work best in the HUNT One Health study. QIAGEN’s Blood and Tissue kit performed on the QiaSymphony platform achieved the highest sensitivity and fulfilled our needs for extraction method. In addition, we found 20 million PE reads sufficient to perform robust taxonomic estimates of the metagenomes, as in line with the findings of Hillmann *et al.* (2018)[59]. This was true for all fecal metagenomes, regardless of animal species, even though we were only able to classify ~10% of the reads from the fecal metagenomes of equines, bovines and sheep. The low classification rate is most probably due to underrepresentation of these reads in the public databases [60,61]. The richness and evenness of these three species’ fecal metagenomes was nevertheless higher than for the canine fecal metagenomes, despite that more reads were classified from the canines. However, the Shannon diversity rapidly tapered off after one million reads for all samples, including the herbivores (Fig 4). Therefore, we are confident that the offered sequencing depth at 20 million PE reads is sufficient for full taxonomic characterization.

For the functional classification, 20 million PE reads are enough to map out the most common functional pathways, but the method has too low sensitivity to detect the abundance of rarer traits. For instance, we could only detect “Beta-Lactamase Class C and other penicillin binding proteins” in samples with more than 5 million reads, and then only 100 reads assigned to this function. In addition, this function is a “bucket” for many closely related ARG genes, and it thus suggests that for the analysis of specific ARG genes we need more sequencing effort to obtain values that can be used for testing experimental differences. This is also in line with other published studies; for instance, 80 million reads were used to do comparative studies on the abundance and type of AMR genes in pigs and chickens [4]. Our dataset is however well fit for qualitative detection, but careful interpretation is necessary as low abundance AMR genes are likely to go undetected. The Norwegian AMR situation is favorable, so low levels of AMR genes are expected in this material.

## Supporting information

S1 Methods **Methodology**: DNA extraction.
(DOCX)

S1 Table DNA extractions. Average DNA concentrations and yield across protocols and species.
(DOCX)

S2 Table Sequencing and annotation statistics. Sample overview of PE read numbers for clean reads, taxonomic classifications with Kraken 2 and Functional classifications with Superfocus.
(DOCX)

S1 Fig Bar chart of the community composition. The eight bacterial species in the mock community and their relative abundance in the ten mock community samples extracted and sequenced using 16S rRNA amplicon technique on the Oxford MinION were visualized in a bar chart. The theoretical composition in terms of 16S rRNA gene abundance as given by the producer, calculated from theoretical genomic DNA composition with the following formula: 16S rRNA gene copy number = total genomic DNA (g) × unit conversion constant (bp/g)/ genome size (bp) × 16S copy number per genome, is 95.9% *Listeria monocytogenes*, 2.8% *Pseudomonas aeruginosa*, 1.2% *Bacillus subtilis*, 0.069% *Escherichia coli*, 0.07% *Salmonella enterica*, 0.012% *Lactobacillus fermentum,* 0.00089% *Enterococcus faecalis*, and 0.000089% *Staphylococcus aureus* (Zymobiotics Research Corpooration, USA). Negative controls were DBS paper only, subjected to the same buffers and procedures as the samples containing fecal material, and is referred to as “blank”.
(DOCX)

S2 Fig Functional classifications of metagenomic reads to the SEED subsystem level 2 category ”Resistance to antibiotics and toxic compounds”. Classification counts were normalized as reads per million reads. Subsystems classes with less than 100 reads were grouped under the category “Subsystems =< 100 reads” to reduce the number of classes in the graph.
(DOCX)

S3 Fig Abundance of the SEED subsystem function ”Beta-lactamase_class_C_and_other_penicillin_binding_proteins” with increasing sequencing effort. The abundance of the function was determined for each subsample metagenome and average for all five samples per sample type. Standard deviation is indicated with error bars. The red dotted line is a cut-off indicating 100 reads. This shows that this function was only detected in the animal microbiomes with more than 100 reads when the sequencing effort was 5 million reads or more.
(DOCX)
