# Supplementary material for: DNA extraction protocols for animal fecal material on blood spot cards
Source: PLoS One. 2025 May 12;20(5):e0313808. doi: 10.1371/journal.pone.0313808 (PMC12068730; doi:10.1371/journal.pone.0313808)
Supplement: S1 Methods — Methodology: DNA extraction. (DOCX) [file pone.0313808.s001.docx]

### S1 Methods:

### Methodology: DNA extraction

#### DNA Extraction Protocol 1 (DNA_P1) and DNA_P2

From each sample, microbial gDNA was extracted from the punches using the semi-automated purification protocol QIAamp® PowerFecal® Pro DNA kit (cat. no 938036, Qiagen) that was run on the Qiasymphony. Inhibitors were removed prior to automated gDNA purification using silica magnetic bead on the QIAsymphony instrument according to the following protocol: 800 µl of Solution CD1 from the QIAamp PowerFecal Pro kit was added, and the sample homogenized during six rounds of 60s each at 6m/s on the FastPrep-24™ Classic instrument (cat number 116004500, MP Biomedicals, USA), allowing samples to cool down on ice for three to four minutes between each round. Finished homogenized samples were quickly spin down at 15000xg for 1min, and the supernatant was transferred to a 2ml microcentrifuge tube and, for DNA_P1, 30ul of proteinase K (>600 mAU/ml) was added and left to work for 15 min at 56°C before 300ul of inhibitor removal buffer CD2 was added. The sample was centrifuged at 15000xg for 1 min, and 600ul of the supernatant was transferred to a clean 2ml Eppendorf tube. Thereafter, DNA was extracted on the QIAsymphony® SP instrument using the QIAsymphony® PowerFecal® Pro DNA Kit (cat. no. 938036, Qiagen) using the DNASoilStool_600_V1 protocol, resulting in 110 µl eluate from each sample.

#### DNA_P3

From each sample, microbial gDNA was extracted from 4x8mm punches using the manual protocol for ZymoBiomics™ DNA Miniprep Kit (Zymo Research Corp., Irvine, CA, USA). In short, 750 µl of lysis solution was added to each of PowerBead Pro Tubes containing the DBS card pieces before homogenizing using the FastPrep-24™ Classic at 6m/s for 60s. x six rounds. The remaining protocol was identical to the instructions provided by the producer for the ZymoBIOMICS DNA Miniprep Kit Cat no D3400T, D4300 and D4304 v 1.4.0 (Zymo Research Corp., Irvine, CA, USA).

#### DNA_P4

From each sample, microbial gDNA was extracted from 4x8mm punches using the automated protocol for MagNA Pure 96 DNA and Viral NA Small Volume Kit on the MagNA Pure 96 Instrument (Roche) with (DNA_P4) an extra proteinase K pretreatment step and standard buffers, according to manufacturer’s recommendations.
