# Supplementary material for: DNA extraction protocols for animal fecal material on blood spot cards
Source: PLoS One. 2025 May 12;20(5):e0313808. doi: 10.1371/journal.pone.0313808 (PMC12068730; doi:10.1371/journal.pone.0313808)
Supplement: S1 Table — Average DNA concentrations and yield across protocols and species. (DOCX) [file pone.0313808.s002.docx]

**S1 Table** Average DNA concentrations and yield across protocols and species.

| Source | DNA extraction method | DNA concentration (ng/µl) | DNA yield (µg) |
| --- | --- | --- | --- |
| Mock | DNA_P1 | 0.7 (SD 0.13) | 0.04 (SD 0.03) |
|  | DNA_P2 | 0.36 (SD 0.3) | 0.11 (SD 0.02) |
|  | DNA_P3 | 0.32 (SD 0.11) | 0.03 (SD 0.01) |
|  | DNA_P4 | 0.11 (SD 0) | 0.01 (0) |
|  | All | 0.4 (SD 0.3) | 0.04 (SD 0.04) |
| Pig | DNA_P1 | 60.2 (SD 21.8) | 4.6 (SD 0.9) |
|  | DNA_P2 | 42.1 (SD 7.8) | 5.1 (SD 4.6) |
|  | DNA_P3 | 16.6 (SD 0.7) | 1.7 (SD 0.07) |
|  | DNA_P4 | 8.5 (SD 0.8) | 0.63 (SD 0.2) |
|  | All | 31.8 (SD 23.6) | 2.5 (SD 2.3) |
| Mock | DNA_P1 | 1.1 (SD 0.15) | 0.1 (SD 0.0) |
| Bovine | DNA_P1 | 13.0 (SD 1.1) | 1.4 (SD 0.13) |
| Dog | DNA_P1 | 4.9 (SD 3.2) | 0.54 (SD 0.4) |
| Horse | DNA_P1 | 10.9 (SD 3.9) | 1.2 (SD 0.4) |
| Sheep | DNA_P1 | 6.2 (SD 3.3) | 0.74 (SD 0.31) |
| Pig | DNA_P1 | 14.5 (SD 4.4) | 1.6 (SD 0.50) |
