# Supplementary material for: DNA extraction protocols for animal fecal material on blood spot cards
Source: PLoS One. 2025 May 12;20(5):e0313808. doi: 10.1371/journal.pone.0313808 (PMC12068730; doi:10.1371/journal.pone.0313808)
Supplement: S2 Table — Sample overview of PE read numbers for clean reads, taxonomic classifications with Kraken 2 and Functional classifications with Superfocus. (DOCX) [file pone.0313808.s003.docx]

**S2 Table:** Sample overview of paired-end read numbers for clean reads, taxonomic classifications with Kraken 2 and Functional classifications with Superfocus.

| **Treatment** | **Dataset** | **PE-reads** | **Kraken2 Taxonomic classifications** | **SuperFocus Functional classifications** |
| --- | --- | --- | --- | --- |
| Blank | DNA_H1H_18_G3 | 396065 | 194449 | 149114 |
| Blank | DNA_H1H_27_G6 | 23390515 | 1910226 | 6514696 |
| Blank | DNA_H1H_40_G12 | 570037 | 234901 | 223689 |
| Blank | DNA_H1H_48_G6 | 1479060 | 426537 | 508077 |
| Blank | DNA_H1H_49_G6 | 462688 | 135788 | 147616 |
| Mock | DNA_H1H_25_H6 | 19534727 | 4206663 | 11063252 |
| Mock | DNA_H1H_40_H12 | 23652500 | 4966244 | 13722491 |
| Mock | DNA_H1H_45_H6 | 23357330 | 4789507 | 13415979 |
| Mock | DNA_H1H_49_H6 | 23316599 | 4833109 | 13448624 |
| Mock | DNA_H1H_5_H6 | 18863241 | 4307751 | 10952255 |
| Bovine | DNA_H1H_14_B3 | 23744149 | 676470 | 5161543 |
| Bovine | DNA_H1H_17_F1 | 23664087 | 613844 | 5308514 |
| Bovine | DNA_H1H_19_B3 | 15450064 | 632693 | 3593304 |
| Bovine | DNA_H1H_34_G9 | 23269869 | 790493 | 5992181 |
| Bovine | DNA_H1H_43_C5 | 18754615 | 485436 | 4004208 |
| Canine | DNA_H1H_1_A6 | 18444980 | 2525127 | 6689077 |
| Canine | DNA_H1H_10_B2 | 23404109 | 2728415 | 8679513 |
| Canine | DNA_H1H_27_E3 | 6104772 | 1058051 | 2516536 |
| Canine | DNA_H1H_28_G5 | 16886409 | 2338809 | 7305861 |
| Canine | DNA_H1H_40_G10 | 19526830 | 2454990 | 6442765 |
| Equine | DNA_H1H_15_G7 | 23630293 | 588592 | 4610045 |
| Equine | DNA_H1H_17_E3 | 22292509 | 625226 | 4175650 |
| Equine | DNA_H1H_32_A9 | 22770346 | 611819 | 4313121 |
| Equine | DNA_H1H_4_G4 | 18838169 | 2149699 | 7309580 |
| Equine | DNA_H1H_44_E2 | 23824639 | 591269 | 4185608 |
| Pig | DNA_H1H_11_A5 | 23913283 | 3671549 | 8125095 |
| Pig | DNA_H1H_11_B5 | 22594427 | 1275181 | 4209698 |
| Pig | DNA_H1H_14_G3 | 23375275 | 1021249 | 5935749 |
| Pig | DNA_H1H_19_C6 | 22810127 | 1308047 | 6579703 |
| Pig | DNA_H1H_30_E5 | 11994137 | 1657448 | 3529784 |
| Sheep | DNA_H1H_36_F6 | 15440326 | 615944 | 3071966 |
| Sheep | DNA_H1H_4_B5 | 18979259 | 608254 | 2818891 |
| Sheep | DNA_H1H_41_A2 | 21541193 | 559879 | 4654686 |
| Sheep | DNA_H1H_44_A1 | 23449904 | 612962 | 5432730 |
| Sheep | DNA_H1H_47_G7 | 23551707 | 642604 | 5152237 |
