# Supplementary material for: DNA extraction protocols for animal fecal material on blood spot cards
Source: PLoS One. 2025 May 12;20(5):e0313808. doi: 10.1371/journal.pone.0313808 (PMC12068730; doi:10.1371/journal.pone.0313808)

**S1 Fig:** **Bar chart of the community compositio**n of the eight bacterial species in the mock community and their relative abundance in the ten mock community samples extracted and sequenced using 16S rRNA amplicon technique on the Oxford MinION. The theoretical composition in terms of 16S rRNA gene abundance as given by the producer, calculated from theoretical genomic DNA composition with the following formula: 16S rRNA gene copy number = total genomic DNA (g) × unit conversion constant (bp/g) / genome size (bp) × 16S copy number per genome, is 95.9% *Listeria monocytogenes*, 2.8% *Pseudomonas aeruginosa*, 1.2% *Bacillus subtilis*, 0.069% *Escherichia coli*, 0.07% *Salmonella enterica*, 0.012% *Lactobacillus fermentum,* 0.00089% *Enterococcus faecalis*, and 0.000089% *Staphylococcus aureus* (Zymobiotics Research Corpooration, USA). Negative controls were DBS paper only, subjected to the same buffers and procedures as the samples containing fecal material, and is hereafter referred to as “blank”.


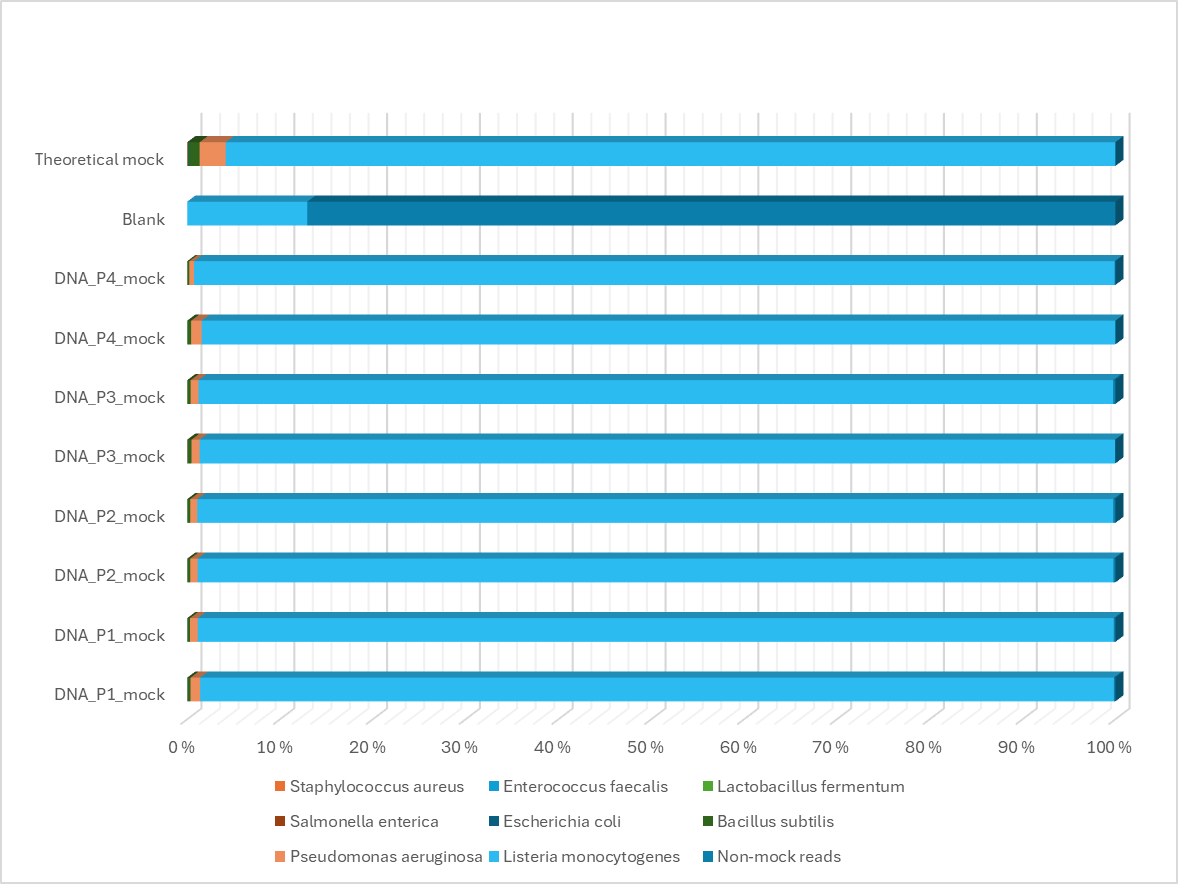

Supplement: S1 Fig — The eight bacterial species in the mock community and their relative abundance in the ten mock community samples extracted and sequenced using 16S rRNA amplicon technique on the Oxford MinION were visualized in a bar chart. The theoretical composition in terms of 16S rRNA gene abundance as given by the producer, calculated from theoretical genomic DNA composition with the following formula: 16S rRNA gene copy number = total genomic DNA (g) × unit conversion constant (bp/g)/ genome size (bp) × 16S copy number per genome, is 95.9% Listeria monocytogenes, 2.8% Pseudomonas aeruginosa, 1.2% Bacillus subtilis, 0.069% Escherichia coli, 0.07% Salmonella enterica, 0.012% Lactobacillus fermentum, 0.00089% Enterococcus faecalis, and 0.000089% Staphylococcus aureus (Zymobiotics Research Corpooration, USA). Negative controls were DBS paper only, subjected to the same buffers and procedures as the samples containing fecal material, and is referred to as “blank”. (DOCX) [file pone.0313808.s004.docx]
