# Supplementary material for: DNA extraction protocols for animal fecal material on blood spot cards
Source: PLoS One. 2025 May 12;20(5):e0313808. doi: 10.1371/journal.pone.0313808 (PMC12068730; doi:10.1371/journal.pone.0313808)
Supplement: S2 Fig — Classification counts were normalized as reads per million reads. Subsystems classes with less than 100 reads were grouped under the category “Subsystems =< 100 reads” to reduce the number of classes in the graph. (DOCX) [file pone.0313808.s005.docx]

**S2 Fig:** **Functional classifications of metagenomic reads to the SEED subsystem level 2 category** ”Resistance to antibiotics and toxic compounds”. Classification counts were normalized as reads per million reads. Subsystems classes with less than 100 reads were grouped under the category “Subsystems =< 100 reads” to reduce the number of classes in the graph.
