# Supplementary material for: DNA extraction protocols for animal fecal material on blood spot cards
Source: PLoS One. 2025 May 12;20(5):e0313808. doi: 10.1371/journal.pone.0313808 (PMC12068730; doi:10.1371/journal.pone.0313808)

**S3 Fig: Abundance of the SEED subsystem function” Beta-lactamase_class_C_and_other_penicillin_binding_proteins” with increasing sequencing effort.** The abundance of the function was determined for each subsample metagenome and average for all five samples per sample type. Standard deviation is indicated with error bars. The red dotted line is a cut-off indicating 100 reads. This shows that this function was only detected in the animal microbiomes with more than 100 reads when the sequencing effort was 5 million reads or more.


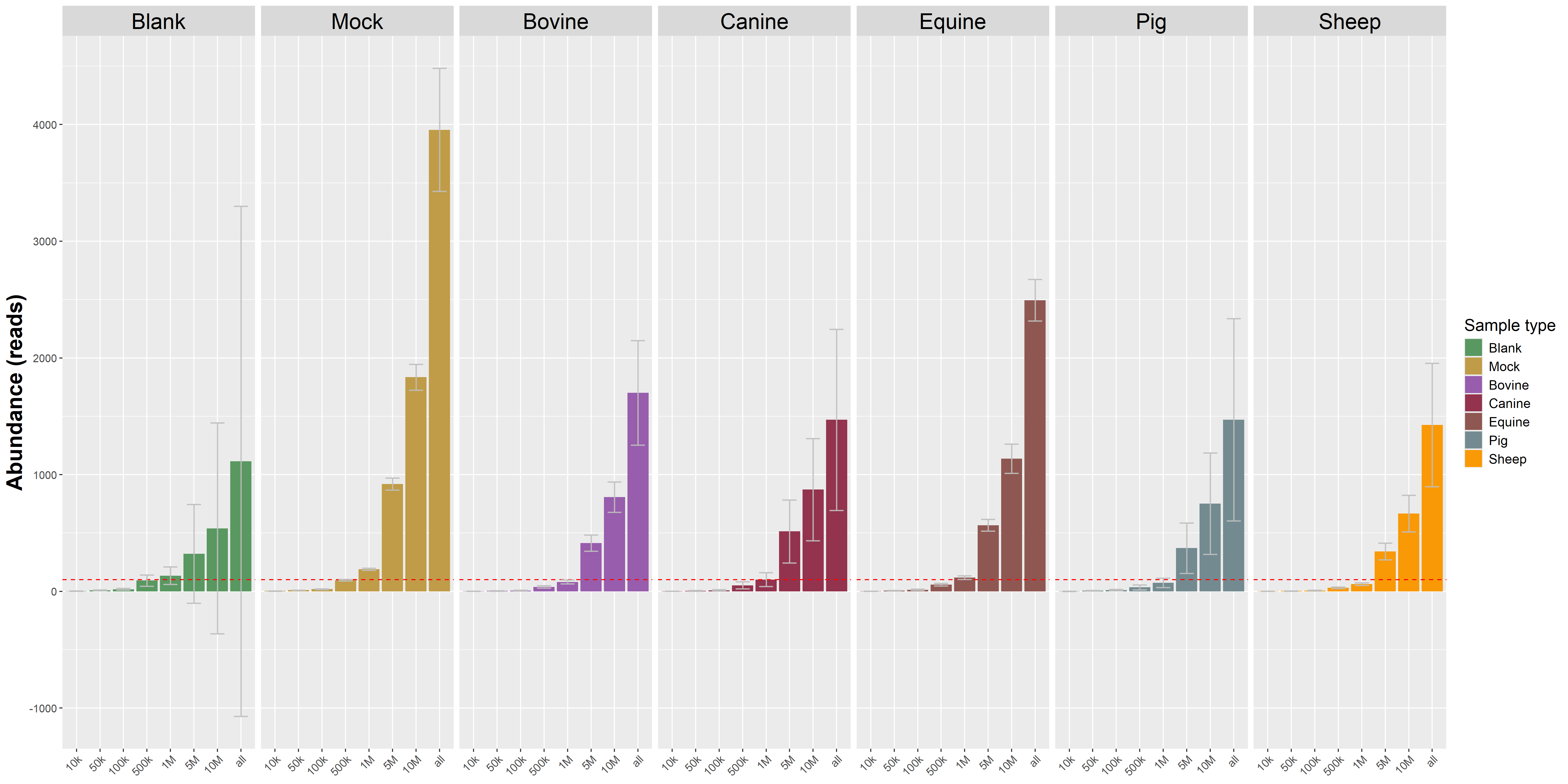

Supplement: S3 Fig — The abundance of the function was determined for each subsample metagenome and average for all five samples per sample type. Standard deviation is indicated with error bars. The red dotted line is a cut-off indicating 100 reads. This shows that this function was only detected in the animal microbiomes with more than 100 reads when the sequencing effort was 5 million reads or more. (DOCX) [file pone.0313808.s006.docx]
